# Nonlinear mixed-effects modelling for single cell estimation: when, why, and how to use it

**DOI:** 10.1186/s12918-015-0203-x

**Published:** 2015-09-04

**Authors:** Markus Karlsson, David L.I. Janzén, Lucia Durrieu, Alejandro Colman-Lerner, Maria C. Kjellsson, Gunnar Cedersund

**Affiliations:** Department of Biomedical Engineering, Linköping University, Linköping, SE-58185 Sweden; Department of Clinical and Experimental Medicine, Linköping University, Uppsala, SE-58185 Sweden; Instituto de Fisiología, Biología Molecular y Neurociencias, Consejo Nacional de Investigaciones Científicas y Técnicas and Facultad de Ciencias Exactas y Naturales, Universidad de Buenos Aires, Buenos Aires, Argentina; Pharmacometrics Group, Pharmaceutical Biosciences, Uppsala University, Uppsala, SE-75124 Sweden; Current Address: Biomedical and Biological Systems Laboratory, School of Engineering, University of Warwick, Coventry, CV4 7AL UK; Modeling and Simulation, AstraZeneca, Mölndal Sweden; Department of Systems and Data Analysis, Fraunhofer-Chalmers Centre, Chalmers Science Park, Gothenburg, SE-412 88 Sweden; IKE, Linköping University, Linköping, 58185 Sweden

**Keywords:** Nonlinear mixed-effects modelling, NLME, Single cell modelling, Singe cell analysis, FRAP

## Abstract

**Background:**

Studies of cell-to-cell variation have in recent years grown in interest, due to improved bioanalytical techniques which facilitates determination of small changes with high uncertainty. Like much high-quality data, single-cell data is best analysed using a systems biology approach. The most common systems biology approach to single-cell data is the standard two-stage (STS) approach. In STS, data from each cell is analysed in a separate sub-problem, meaning that only data from the same cell is used to calculate the parameter values within that cell. Because only parts of the data are considered, problems with parameter unidentifiability are exaggerated in STS. In contrast, a related approach to data analysis has been developed for the studies of patient-to-patient variations. This approach, called nonlinear mixed-effects modelling (NLME), makes use of all data, when estimating the patient-specific parameters. NLME would therefore be advantageous compared to STS also for the study of cell-to-cell variation. However, no such systematic evaluation of the two approaches exists.

**Results:**

Herein, such a systematic comparison between STS and NLME has been performed. Different examples, both linear and nonlinear, and both simulated and real experimental data, have been examined. With informative data, there is no significant difference in the results for either parameter or noise estimation. However, when data becomes uninformative, NLME is significantly superior to STS. These results hold independently of whether the loss of information is due to a low signal-to-noise ratio, too few data points, or a bad input signal. The improvement is shown to come from both the consideration of a joint likelihood (JLH) function, describing all parameters and data, and from an *a priori* postulated form of the population parameters. Finally, we provide a small tutorial that shows how to use NLME for single-cell analysis, using the free and user-friendly software Monolix.

**Conclusions:**

When considering uninformative single-cell data, NLME yields more accurate parameter and noise estimates, compared to more traditional approaches, such as STS and JLH.

**Electronic supplementary material:**

The online version of this article (doi:10.1186/s12918-015-0203-x) contains supplementary material, which is available to authorized users.

## Background

Cell-to-cell variation is one of the most intriguing and important fields in today’s cell biology research. Historically, the fact that cells are different from each other has been neglected, and this neglect has led to erroneous conclusions and descriptions of the system [1]. Within the systems biology community, modelling of cells has typically been performed based on data from the average of cells, and the model has thus described an average cell. In several well-known cases, this average cell has turned out to be highly non-representative of the true underlying cellular behaviour. For instance, the prevailing view of the signalling cascade involving Casp-3 was that the changes were described as gradual, since this was the average population behaviour; however, this population behaviour was obtained by a gradual change in the number of cells that had switched from one state to another, where the switch in each individual cell was fast [2]. Cell-to-cell variation is also at the heart of understanding cell differentiation, which involves the important special cases of stem cell research and research on the development of tumours [3].

**Fig. 1:**
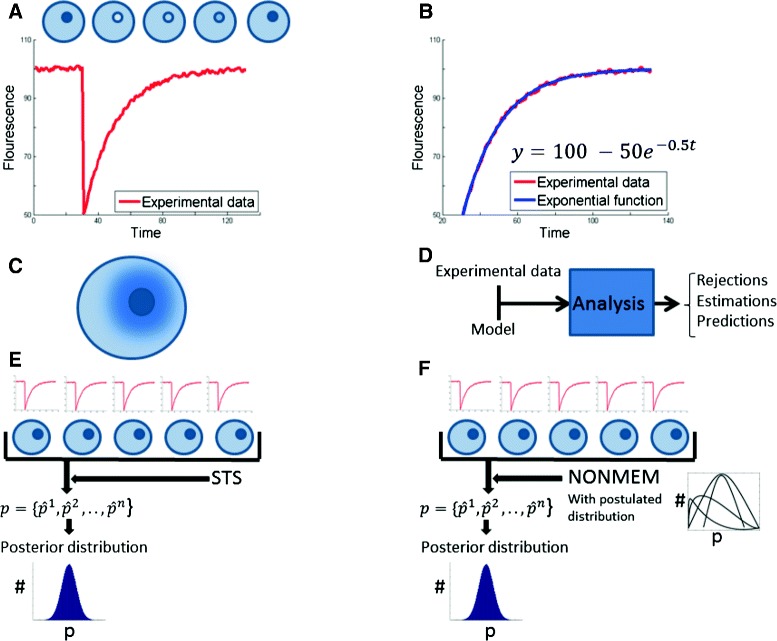
Different approaches to single-cell analysis based on FRAP data. **a** The basic principle behind FRAP experiments: a part of a cell is bleached, and the recovery is followed. **b** The most common analysis of FRAP data: to fit an exponential function to the data. **c** PDE simulations, where the gradients are continuous in the cytosol. **d** The reversed-engineering approach to FRAP data, to draw conclusions in terms of model rejections and estimation of parameters and predictions. **e** The STS approach: fit a model to each data separately, and then combine the estimations to get the distributions. **f** The NONMEM approach: to add a postulated distribution for the parameter distributions among the population, and then fit to all the data at the same time using a joint likelihood function

One common example of single cell data is fluorescent recovery after photo-bleaching (FRAP), which examines the time-dependent response to the bleaching of a part of a cell (Fig. 1a). This response normally follows an exponential decline/increase. The most straightforward analysis of such data is therefore to simply fit an exponential curve to the data, and evaluate the value of the exponent [4] (Fig. 1b). The limitation of such an approach is that the exponent does not correspond to the velocity of any specific mechanism, but to a phenomenological description lumping many sub-processes together. A more mechanistically interpretable approach is to form a model based on prior knowledge of the underlying sub-processes. Sometimes such a model is formulated using partial differential equations (PDEs) [5] (Fig. 1c). The limitation of PDEs is that a single simulation is very computationally time-consuming. Therefore, PDE-based models are usually utilized for forward-simulation, i.e. where simulations of different scenarios are performed, but where the model is assumed as known. Another important type of modelling is known as reversed-engineering, in which parameters with mechanistic interpretation are estimated based on the data, and where conclusions can be drawn regarding mechanisms in the biological system [6–9] (Fig. 1d).

Such reverse-engineering approaches to research on cell-to-cell variation have typically been pursued using ordinary differential equations (ODEs) and a method known as the standard two-stage approach (STS) (Fig. 1e) [10]. STS is the typical approach used to study ODEs in systems biology [7], applied to the problem of single-cell characterization. In other words, after a non-rejected model has been chosen, the parameters are determined in each cell separately (stage 1 in STS). Thereafter, the distribution of the cell population’s parameters are compared and determined (stage 2). One of the problems with STS is that the data in one cell alone may be insufficient to determine the individual parameters for that particular cell accurately, i.e. that the uncertainty in each of the individual parameters are unacceptably high [11]. In such situations, it may be beneficial to make use of the data and information that exists also for the other cells.

Such approaches, where one problem for one unit is solved in connection to the corresponding problem for all other units, have been developed in various other disciplines, under a variety of names. One such name is multi-task learning. This name is used in the machine learning community, and has been successfully applied to e.g. classification, pattern recognition, etc [12–16]. However, multi-task learning approaches seem only rarely to have been applied to the task of estimating parameters in an ODE [17], and not at all to cell-to-cell variation studies. In contrast, the same idea has also been developed under the name mixed-effects modelling, and the sub-class known as nonlinear mixed-effects modelling (NLME) (Fig. 1f) has been widely used to estimate parameters in ODEs [10, 11]. The majority of NLME applications appear within the field of pharmacokinetics, i.e. for models that describe the uptake, breakdown, and effect of a drug in human or animal subjects.

Regarding cell-to-cell variation, there are a few recent examples that make use of NLME, but there is no systematic evaluation of when and why NLME is advantageous compared to STS. One important series of papers regarding NLME and cell-to-cell variation have been published by Zechner et al. The first such papers were applied to snapshot data, i.e. data were only a single data point is available for each cell [18–20]. Recently, this approach has been generalized to also be able to deal with time-series data [21]. The Zechner papers focus on issues related to noise, and for instance seek to differentiate between extrinsic and intrinsic noise. Presumingly for this reason, they work exclusively with continous-time markov chains (CTMC), and do not present any results for ODEs, despite the fact that ODEs is the most widely used model class in the systems biology community [22]. In other words, the Zechner papers do not explain *how* to use NLME to study cell-to-cell variations using ODEs. Furthermore, the Zechner papers do not demonstrate or explain *why* or *when* NLME are superior to STS. There is one conference paper on NLME-based ODE-estimation of single-cell data [23]. This paper presents a comparison between such ODE-estimation and an early version of the Zechner snapshot approach [20]. However, also this paper [23] does not explain when or why NLME should be used instead of STS. Herein we present such an explanation.

**Fig. 2:**
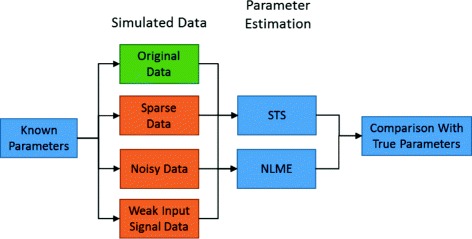
Overview of the analysis in the paper. Both Model 1 and Model 2 are analysed with simulated data. The data comes in four forms: Original, Sparse, Noisy and Weak Input Signal Data. The question is whether the true parameters can be obtained, and whether the non-informative cases (*orange boxes*) show different results from the informative data (*green box*)

More specifically, we demonstrate the occasional importance of studying cell-to-cell variation with NLME rather than using STS. Based on simulated data, where the true model structure and parameter values are known (Fig. 2), we show that for the case of uninformative data, NLME is advantageous over STS regarding parameter estimation: both kinetic and noise parameters were estimated significantly closer to the true values compared to estimating the parameters using STS. We show that this advantage seems independent of the reason for the lack-of-information in the data, and also unravel where the advantage comes from. We finally also demonstrate that NLME can be used for the analysis of real experimental FRAP data from the yeast *Saccharomyces cerevisiae*.

## Methods

### The standard two stage approach

In STS, the following model structure is used

(1) $$ \dot{x}^{i} = f\left(x^{i},u^{i},p^{i}\right)   $$

(2) $$ y^{i} = h\left(x^{i},u^{i},p^{i}\right)   $$

where *x*^*i*^ is the state vector for the *i*:th individual; *u*^*i*^ is the input signal vector for individual *i*; *p*^*i*^ is the parameter vector for the *i*:th individual; *f* and *g* are nonlinear vector functions; and *y*^*i*^ is the vector of observations for individual *i*.

#### Parameter estimation

In Stage 1, the parameters for each individual *p*_*i*_ are estimated. Note that in a modelling framework such as eqs. (1)-(2), no information is shared between the individuals, which makes the parameter estimation problem for each individual a separate estimation problem. In Stage 2, the variability of the parameter estimates are calculated (Fig. 1e).

In the case of only estimating the parameters, we minimize the following cost function, based on the sum of squares of the residuals

(3) $$ cost_{\chi^{2}}(p) = \sum^{n_{y}}_{j=1}\sum^{N}_{i=1}\frac{\left(y_{ij}-\hat{y}_{ij}(p)\right)^{2}}{\sigma_{ij}^{2}}   $$

where *i* and *j* denotes the *i*:th observation in the *j*:th state; *y*_*ij*_ is the experimental data; $\hat {y}_{\textit {ij}}$ is the corresponding simulated output from the model; and *σ*_*ij*_ is the standard deviation of the experimental measurement. In other words, the kinetic parameters *p* are given by

(4) $$ \hat{p} = \arg\min\left[cost_{\chi^{2}}(p)\right]   $$

Note that this approach cannot be used to estimate *σ*, since in eq. (3) *σ* only appears in the denominator, and the optimum for *σ* therefore lies at +*∞*. For estimation of the noise, we therefore use the more general approach of maximizing the full log-likelihood function

(5) $$ \begin{aligned}  -l(p,\sigma) &= \sum^{n_{y}}_{j=1}\sum^{N}_{i=1}\frac{\left(y_{ij}-\hat{y}_{ij}(p)\right)^{2}}{\sigma_{ij}^{2}} + \sum^{n_{y}}_{j=1}\sum^{N}_{i=1}log\left(\sigma_{ij}^{2}\right)\\ & \quad+ n_{y}Nlog(2\pi)  \end{aligned}  $$

where

(6) $$ ({\hat p,\hat \sigma}) = \textrm{arg max}\left[l(p,\sigma)\right]   $$

where *σ* is the vector of all *σ*_*ij*_, which now normally have their optima different from 0 or infinity, since they appear both in the numerator and in the denominator.

#### Software

Modelling and parameter estimation with the STS approach was performed using MATLAB (Mathworks). The simulation of the model was performed with the function SPBDsimulate in The Systems Biology Toolbox2 (SBTB2) (sbtoolbox2.org). Optimisation was done both using the built-in local optimisation algorithm *lsqnonlin* (which uses Gauss-Newton methods), and using the global simulated annealing and nonlinear simplex approach available in SBTB2. All simulations and optimisations, except the ones made for the noise estimation and the JLH were done using a PC (Processor: Intel Core i5-3470 3.20 GHz, Memory: 8.00 GB, manufacturer: Hewlett-Packard). The simulations and optimisations for the noise estimation and the JLH were done using a laptop (Processor: Intel Core i5-560M 2.667 GHz, Memory: 2x 2048 MB, manufacturer: Samsung, DDR3-10600S, 1333 MHz). All MATLAB-files used (including datasets) are available in Additional file 1.

### The nonlinear mixed-effects approach

NLME is a general modelling approach that can be applied to analyse any type of system that can be described by eqs. (7)-(11), i.e. to a system that is made up of individuals belonging to a joint population, and where the individuals’ parameter values belong to the parameter distribution of the population. This link between the parameter values among the individuals allows information to be shared between the individuals. The idea is that this information sharing may result in better estimates for both the individual parameters and for the covariance matrices. More specifically, in NLME models, the following general model structure is used

(7) $$\begin{array}{@{}rcl@{}} \dot{x}^{i} & = & f\left(x^{i},u^{i},\phi^{i}\right)  \end{array} $$

(8) $$\begin{array}{@{}rcl@{}} y^{i} & = & h\left(x^{i},u^{i},\phi^{i},\varepsilon^{i}\right)  \end{array} $$

(9) $$\begin{array}{@{}rcl@{}} \phi^{i} & = & g\left(\Theta,\eta^{i},Z^{i}\right) \end{array} $$

(10) $$\begin{array}{@{}rcl@{}} \eta^{i} &\in& N(0,\Omega)  \end{array} $$

(11) $$\begin{array}{@{}rcl@{}} \varepsilon^{i}&\in& N(0,\Sigma)  \end{array} $$

where *x*^*i*^, *u*^*i*^, and *y*^*i*^ are, just as for STS, the state, input, and measurement vectors for individual *i*; where *ϕ*^*i*^ is the parameter vector for the *i*:th individual, which now no longer is a free variable, but instead depends on *Θ*, the population parameter vector describing the typical individual in the population, *Z*^*i*^ the covariates (not used in this paper), and *η*^*i*^, the random effects; and where *Ω* and *Σ* describes the covariance matrices of the random effects, *η*^*i*^, and the measurement noise, *ε*^*i*^, respectively.

#### Parameter estimation

In NLME there are two types of parameters to estimate: the fixed effects, *Θ*, and the variances of the random effects, *Ω* and *Σ*.

The fixed effects describe the main trend, i.e. the typical value of the model parameters. For a single-cell model, such fixed effects could be the typical population value of a kinetic parameter.

There are two types of random effects; between-cell, *η*, and between-sample, *ε*, variability. The between-sample variabilities are related to the residuals, in that they describe the differences between the predicted, $\hat {y}$, and the observed, *y*, measurement values. In practice, the software packages used herein, NONMEM and Monolix, can handle e.g. additive

(12) $$ y = \hat{y} + \varepsilon   $$

proportional, and a combination of additive and proportional between-sample variability

(13) $$ y = \hat{y} \cdot (1 + \varepsilon_{1}) + \varepsilon_{2}   $$

The effect of the between-cell random effects, *η*, on the cell-specific parameters, *ϕ*^*i*^, can in principle be described by any function, *g*, and the used software packages support both normal, log-normal, and user-specified distributions (specific examples are provided in the two test cases below).

The marginal likelihood (L) of the model parameters for the data is the product of the individual marginal likelihoods of the cells, *j* according to

(14) $$ \begin{aligned} P(y^{\,j}|\Theta,\Sigma,\Omega) &= L^{j}\left(\Theta,\Sigma,\Omega|y^{\,j}\right) = \int{P(y^{\,j},\eta^{\,j}|\Theta,\Sigma,\Omega)d\eta^{\,j}} \\ & = \int{P\left(y^{\,j}|\eta^{\,j},\Theta,\Sigma\right) \cdot P\left(\eta^{\,j}|\Omega\right)d\eta^{\,j}}  \end{aligned}  $$

(15) $$ \begin{aligned} L^{j}(\Theta) &= \int^{+\infty}_{-\infty} {\left(\frac{1}{\sqrt{2\pi\Sigma}}\right)^{m}e^{-\frac{\sum\limits_{j=1}^{\eta^{\,j}} \left(y^{j}-\hat{y}^{\,j}\right)^{2}}{\Sigma}}}\frac{1}{\sqrt{2\pi\Omega}}e^{-\frac{\eta^{{\,j}^{2}}} {\Omega}}d\eta^{j} \\ &= \int^{+\infty}_{-\infty}{h^{\,j}(\eta,\theta)d\eta^{\,j}}  \end{aligned}  $$

Where *m* is the number of observations per cell, i.e., *m* =*N*·*n*_*y*_. The parameters are estimated through minimizing -2 log of the likelihood (-2LL). Since there is no closed form solution to the marginal likelihood various approximations are available. The most commonly used approximation is the first-order conditional estimation method where L is linearised with a first order Taylor expansion around the estimates of the random effects, i.e. around *η*. In the NONMEM example (Model 1), the numerical search for the minimum of the -2LL is implemented according to a modified algorithm by [24] which is a derivate-free quasi-Newton type minimization algorithm. The objective function value (OFV) reported by the software is proportional to -2LL. In the Monolix example (Model 2), the numerical search is done by a stochastic approximation of the expectation maximisation algorithm [25].

### Software

The NLME approach has been implemented in several software packages [26]. In this paper, the two software packages NONMEM and Monolix are both used. This is done to show consistency in terms of results across different software packages, but also as a way of presenting several choices to the reader.

For the analysis with NONMEM version 7.2.0 [27] was used. The interaction with NONMEM is made through NM-TRAN, a language translating user-defined code and datasets into FORTRAN77. ODEs can be defined by the user and for this particular project a differential equation solver, for non-stiff systems, was used (ADVAN6) together with the first-order conditional estimation (FOCE) method. Perl-speaks-NONMEM (PsN)3.5.3 [28, 29] was used for execution of models. NONMEM and PsN were installed on a PC and a laptop, which systems details were the same as described in the section The standard two stage approach, with the fortran compiler GNU gfortran.

For the analysis with Monolix, version 4.3.2 was used, as implemented in MATLAB version 8.1 [30]. Importantly, for users who do not have access to MATLAB, a stand alone version also exists. The models are written in a language called mlxtran, which apart from having a rich library of PKPD-models also allows the users to define their own ODEs [31]. SBTB2 also contains functions for translating SBTB2-models into mlxtran, using the addon-package SBPOP. Compared to NONMEM, Monolix offers a more user friendly environment, including a graphical user interface. For the beginner, we therefore recommend to use Monolix, and we have also developed a small tutorial, which explains how to us it for single-cell models. This tutorial, together with all scripts used to perform the analysis in this paper, is available in the Additional file 1. All the analysis regarding Monolix was performed on the same PC as described in the section The standard two stage approach. All NONMEM-files and Monolix-files used (including datasets) are available in Additional file 1.

### An in-between approach: the joint likelihood function

There are two main differences between STS and NLME which both potentially could lead to improvements in the parameter estimation: i) in NLME one forms a likelihood function for the parameter estimation to the combined data set for all cells, and ii) in NLME one postulates a distribution for the variation of the parameter values across the cell population.

To analyse where the improvement in the parameter estimation originates from, we also did some analysis with an in-between approach: the joint likelihood function (JLH) approach. In JLH, we only use improvement aspect i), the single likelihood function for the combined data set, but do not postulate a joint parameter distribution. In other words, instead of eqs. (5)-(6), we use the following two equations:

(16) $$ \begin{aligned} &-l_{\textrm{JLH}}(p^{1},p^{2}\ldots,\sigma^{1},\sigma^{2},\ldots) = \sum^{N_{c}}_{k=1}\sum^{n_{y}}_{j=1}\sum^{N}_{i=1}\frac{\left(y^{k}_{ij}-\hat{y}^{k}_{ij}(p)\right)^{2}}{\left(\sigma^{k}_{ij}\right)^{2}}\\ &\qquad + \sum^{N_{c}}_{k=1}\sum^{n_{y}}_{j=1}\sum^{N}_{i=1}log\left(\left(\sigma_{ij}^{k}\right)^{2}\right) + N_{c}n_{y}Nlog(2\pi)  \end{aligned}  $$

where

(17) $$ {\fontsize{9.2pt}{9.6pt}\selectfont{\begin{aligned} \left({\hat p^{1},\hat p^{2},\ldots\hat \sigma^{1}, \hat \sigma^{2},\ldots}\right) = \arg \max\left[l_{\textrm{JLH}}\left(p^{1},p^{2} \ldots,\sigma^{1},\sigma^{2}\right), \ldots \right] \end{aligned}}}  $$

where *N*_*c*_ denotes the number of cells.

#### Software

The software and optimization and model formulation tools used for the JLH analysis is the same as for STS.

### Experimental data

The experimental procedures are further discussed in [32]. FRAP experiments were performed using YFP. We used yeast cells of the BY4741 genetic background expressing YFP under the control of the constitutively expressed ACT1 promoter (P _ACT1_-YFP). In addition, this strain expressed two extra fluorescent protein reporters: a CFP-Ace2 fusion and a Myo1-mCherry fusion, both driven by their own promoters. We used Ace2 to locate the nucleus and determine the cells position in the cell cycle (Ace2 is nuclear only at the end of mitosis and early G1), and we used Myo1 to confirm mother-daughter separation (Myo1 forms a ring at the bud-neck during mitosis, which disappears when cytokinesis is complete). In this way, we minimized cell-to-cell variation in our measurements related to cell cycle position, without disturbing the system with synchronization procedures.

We used exponentially growing cells cultured in synthetic medium (BSM-TRP,LEU,URA 2 % glucose). For the experiments, we attached cells to the bottom of 384-well glass bottom plates (MGB101-1-1-LG, Matrical Biosciences, Spokane, Washington, USA). To prevent cells from moving during the experiment, we pre-treated the wells with concanavalin A (type V; Sigma-Aldrich, St. Louis, MO, USA), as previously described (Colman-Lerner 2005).

Photobleaching of individual nuclei was performed using an Olympus IX-81 inverted microscope with a FV1000 confocal module with an oil immersion Olympus UplanSapo 63X objective (numerical aperture, NA 1.35). We used an automatic z-axis control, a motorized x-y stage, a 458-488-515 argon laser, a 543 He-Ne laser and photomultiplier (PMT) Hamamatsu R6353.

For train-of-FRAP experiments, we imaged YFP with the 515 nm laser. In each cell, we repeated four times the following procedure: we first took 5 images, then performed a partial photobleaching (roughly reducing the signal by 50 %), then measured the recovery for 7 sec (30 images, time resolution 0.22 sec). From the photobleaching step, we used a 100 % laser power on a small sub-area of the nucleus (radius 0.25 m) during 0.16 sec. Subsequently, we imaged using 1 % laser power and 4 sec/pixel scanning speed. We set the confocal microscope pinhole to wide open (500 m).

Quantification of total fluorescence in each compartment was performed in ImageJ, by manually drawing regions of interest (ROIs) in the nucleus and the cytosol, and quantifying it using the ImageJ plugin “Time Series Analyzer V2”. A typical ROI was a circle of 200 nm in radius. We applied photobleaching and autofluorescence corrections to all images as described in [32].

### Two case studies: a linear and a nonlinear model

Let us now introduce the two examples considered in this paper. The first, Model 1, is a linear model, describing transport of YFP, and the second, Model 2, is a nonlinear model describing the osmo-regulation of yeast cells.

#### Linear model

**Fig. 3:**
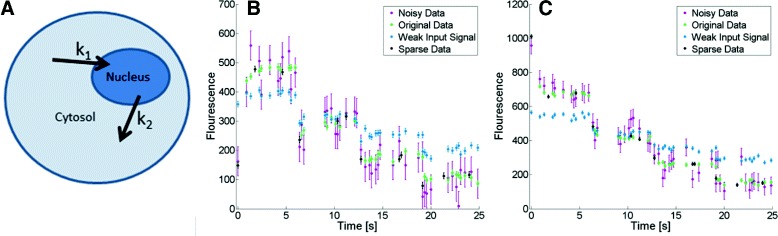
Model 1 and the data. **a** Sketch of Model 1. **b** Example of simulated data of the nucleus under different conditions. **c** Example of simulated data of the cytosol under different conditions

The state-space description of Model 1 is given by the following four equations

(18) $$\begin{array}{*{20}l} \frac{dn}{dt} &= -k_{1}\cdot n + k_{2}\cdot c  \end{array} $$

(19) $$\begin{array}{*{20}l} \frac{dc}{dt} &= k_{1}\cdot n - k_{2}\cdot c  \end{array} $$

(20) $$\begin{array}{*{20}l} y_{n} &= n + \epsilon  \end{array} $$

(21) $$\begin{array}{*{20}l} y_{c} &= c + \epsilon  \end{array} $$

where *n* and *c* are the amounts of YFP in the nucleus and in the cytosol, respectively; where *k*_1_ and *k*_2_ are the transports from and to the nucleus, respectively; and where *y*_*n*_ and *y*_*c*_ are the two measurement signals (Fig. 3b and c). A sketch of the model is given in Fig. 3a.

In STS estimation, data from each cell were analysed separately, potentially yielding as many *k*_1_ values as there were cells in the experiment. For simulation of the data using eqs. (18)-(21), the following equations were used to obtain the initial conditions

(22) $$ n(t_{FRAP,j}) = p_{n,j}\cdot y_{n}(t_{FRAP,j})   $$

(23) $$ c(t_{FRAP,j}) = p_{c,j}\cdot y_{c}(t_{FRAP,j})   $$

where *t*_*FRAP*,*j*_ is the first time point after FRAP *j*. There were four FRAPs, two states, and two kinetic parameters, and thus 10 unknown parameters in the parameter vector *p*

(24) $$ p = (k_{1},k_{2},p_{n,1},p_{n,2},p_{n,3},p_{n,4},p_{c,1},p_{c,2},p_{c,3},p_{c,4})   $$

In the NLME estimation, the individual rate constants ${k_{1}^{j}}$ and ${k_{2}^{j}}$ were described by the following equations

(25) $$ {k_{1}^{\,j}} = \theta_{k_{1}} \cdot e^{\eta_{k_{1}}^{\,j}}   $$

(26) $$ {k_{2}^{\,j}} = \theta_{k_{2}} \cdot e^{\eta_{k_{2}}^{\,j}}   $$

where $\theta _{k_{1}}$ and $\theta _{k_{1}}$ are the typical values of *k*_1_ and *k*_2_ in the cell population, respectively, and $\eta _{k_{1}}^{\,j}$ and $\eta _{k_{2}}^{\,j}$ are random effects describing the difference between the typical and individual values. $\eta _{k_{1}}$ and $\eta _{k_{2}}$ belongs to normal distributions with mean 0 and estimated variances, $\omega _{k_{1}}^{2}$ and $\omega _{k_{2}}^{2}$, respectively. The estimated variances $\omega _{k_{1}}^{2}$ and $\omega _{k_{2}}^{2}$ can be correlated. Both the variances $\omega _{k_{i}}^{2}$ and their correlations are collected in the variance-covariance matrix *Ω*. Thus, five parameters are needed to describe the individual ${k_{1}^{\,j}}$ and ${k_{2}^{\,j}}$ for the entire population: $\theta _{k_{1}}$, $\theta _{k_{2}}$, $\omega _{k_{1}}^{2}$, $\omega _{k_{2}}^{2}$, and the off-diagonal element in *Ω*. Unlike in STS, this number is always five, independent of the number of analysed cells.

The initial conditions were modelled as

(27) $$ n(t_{FRAP,j}) = e^{(\eta_{j}\cdot\Theta_{3})}\cdot y_{n}(t_{FRAP,j})   $$

(28) $$ c(t_{FRAP,j}) =e^{(\eta_{j}\cdot\Theta_{3})}\cdot y_{c}(t_{FRAP,j})   $$

where *η*_*j*_∈*N*_*j*_(0,1) and *Θ*_3_ is the standard error of the residual error.

Finally, the noise is for both STS and NLME assumed to be additive, which also is how the simulated data was generated. In other words, simulations were done for different values of the parameters, additive non-correlated noise was added, and the ability of STS and NONMEM to retrieve the parameter values and the standard deviation of the noise was evaluated.

#### The nonlinear model

**Fig. 4:**
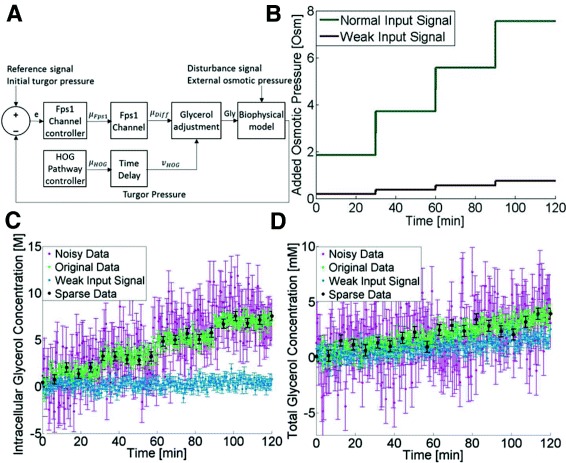
Model 2 and the different inputs and data. **a** Sketch of Model 2. **b** The two different input signals used in the analysis. **c** Artificial data of intracellular glycerol concentration under different conditions **d** Artificial data of total glycerol concentration under different condition

Model 2 is a model published by Gennemark et al. [33] (equations for the model can be found in Additional file 2). The model describes how the yeast cell reacts to an osmolarity shock by producing glycerol via activation of the protein Hog1 (Fig. 4a). A description of all the equations can be found in the Supplementary material. The model consists of 4 ODEs, 10 parameters, and 3 algebraic equations, including several nonlinearities, both products of states, and events switching between two expressions depending on the value of a state. 4 parameters (Ve, kp2, kHOG, td) were optimized from the simulated data, and the remaining 6 parameters were kept at their literature values. The initial conditions were assumed to be known, as was the noise level. The input of the model is the addition of salt to the cells (Fig. 4b) and the measured output signal of the model is intracellular (Fig. 4c) and total (intracellular + extracellular) glycerol concentrations (Fig. 4d).

In the NLME estimation, the model parameters are described by the following equations.

(29) $$\begin{array}{@{}rcl@{}} \text{Ve}^{\,j} & = & \theta_{\text{Ve}} \cdot e^{\eta_{\text{Ve}}^{\,j}}  \end{array} $$

(30) $$\begin{array}{@{}rcl@{}} \textrm{kp2}^{\,j} & =& \theta_{\textrm{kp2}} \cdot e^{\eta_{\textrm{kp2}}^{\,j}} \end{array} $$

(31) $$\begin{array}{@{}rcl@{}} \textrm{kHOG}^{\,j}& =& \theta_{\textrm{kHOG}} \cdot e^{\eta_{\textrm{kHOG}}^{\,j}} \end{array} $$

(32) $$\begin{array}{@{}rcl@{}} \text{td}^{j} & =& \theta_{\text{td}} \cdot e^{\eta_{\text{td}}^{\,j}}  \end{array} $$

where *θ*_*x*_ is the typical value of *x* in the cell population, and ${\eta _{x}^{j}}$ is the random effect describing the difference between the typical and individual values for parameter *x* for cell *j*. These random effects (*η*_Ve_, *η*_kp2_, *η*_kHOG_, and *η*_td_) belongs to normal distributions with mean 0 and estimated variances ($\omega _{\text {Ve}}^{2}$, $\omega _{\textrm {kp2}}^{2}$, $\omega _{\textrm {kHOG}}^{2}$, and *ω*_td_). There are no covariances, i.e. the matrix *Ω* is set to be diagonal. Both measurements of the model, intracellular and total glycerol concentrations, have additive noise

(33) $$\begin{array}{@{}rcl@{}} y_{\text{ic}} & = & \hat{y}_{\text{ic}} + \varepsilon_{\text{ic}}  \end{array} $$

(34) $$\begin{array}{@{}rcl@{}} y_{\text{tot}}& = & \hat{y}_{\text{tot}} + \varepsilon_{\text{tot}} \end{array} $$

where index ic means intracellular, tot means total, and where *ε*_ic_ and *ε*_tot_ are normally distributed, with mean 0 and estimated variances.

#### Comparison of performance

**Fig. 5:**
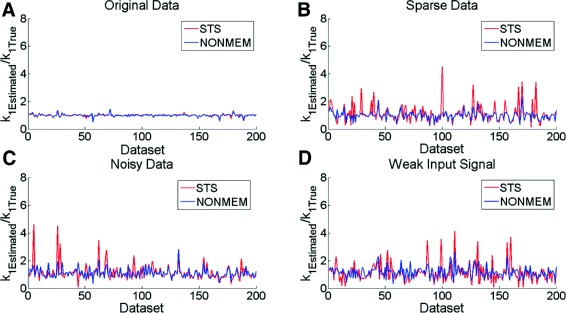
Parameter estimation for Model 1. Analysis in the case of simulated data and parameter estimation for the parameter *k*_1_ in the case of known noise for Model 1 using data that is: **a** under good condition, **b** sparse sampling, **c** noisy, and **d** with a weak input signal respectively. The results are normalized by dividing with the known true value. The x-axes corresponds to the 200 simulated datasets. In comparison, the results from the parameter estimation are similar between STS and NONMEM in the case of the Original Data, but there is a clear advantage of using NONMEM when the quality of the data decreases

The performance of STS and NLME are analyzed by comparing the relative deviation from the true parameter value (estimated parameter/true parameter) (Figs. 5, 7). Apart from figures showing the relative deviation for each parameter for each artificial cell, the differences between STS and NLME is also accompanied by a Student’s t-test. The t-test is pairwise, and tests whether the relative deviation (the distance from 1) is significantly different between STS and NLME.

### Ethics

The experiments and data collection were carried out on *Saccharomyces Cerevisiae*, which has no associated ethical issues.

## Results

### Linear model: NLME is advantageous in cases of low-quality data

We generated FRAP data structured like the real experimental data (Methods) but with known true kinetic parameters, in order to determine whether there is a difference between STS’s and NLME’s ability to estimate the true parameters in a system (Fig. 2). For Model 1, NLME was implemented using the software NONMEM. Both STS and NLME estimated the parameters using the true structural model, which for the case of NLME also included the true additive error model eqs. (20)-(21). In addition, NLME were given the true form of the kinetic parameter distributions among the cell population (that *η* had a log-normal distribution with an unknown mean and standard deviation). The standard deviation of the measurement noise was in the first part of the analysis assumed to be known.

The generated FRAP data were divided into four different cases: Original Data, Sparse Data, Noisy Data, and Weak Input Signal Data (Fig. 3b and c). The Original Data (Fig. 3b and c, green) had 48 observations per state, giving a total of 96 observations per artificial cell. The Sparse Data (Fig. 3b and c, black) had 24 observations per artificial cell (1/4 of the number of observations in the Original Data). The noise of the Noisy Data (Fig. 3b and c, purple) had a standard deviation of 50, compared with 10 for the Original Data. The Weak Input Signal Data had FRAPs that were half of the strength of the FRAPs used to generate the Original Data. The initial conditions were the same for all time-series in the Original, Noisy, and Sparse Data, since they correspond to the same perturbation from the steady state. For the same reason, the Weak Input Signal Data had an initial condition closer to the steady state. All initial conditions were estimated from the simulated datasets eqs. (27)-(28)

In Fig. 5, the results from the estimation of the kinetic parameter *k*_1_ from both STS (red) and NLME (blue) can be seen. From this figure, it is clear that for the case of the Original Data (Fig. 5a), there is no significant difference between STS and NLME in terms of their ability to estimate the true parameters: the relative deviation from the true values were 0.991 ±0.09 for NLME and 0.993 ±0.09 for STS (mean ±SD). However, for the cases when the quality of the data is reduced in either of three different ways (sparseness in Fig. 5b, a larger noise level in Fig. 5c, and a weaker input signal in Fig. 5d), there is a larger difference: STS often fails, while NLME still produces roughly the same results. These differences are also supported by a paired Student’s t-test (*p*<0.05). Similar results can be seen for the other kinetic parameter, *k*_2_, in Figure S1 in Additional file 3.

### Linear model: the same improvement holds for noise estimation

**Fig. 6:**
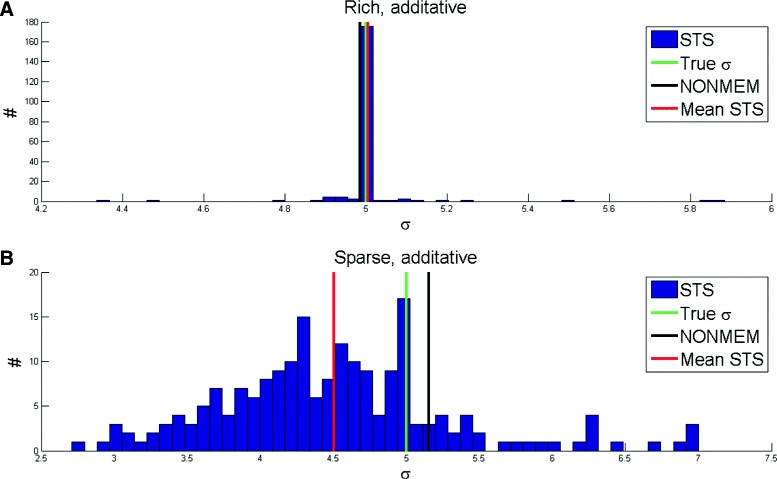
Noise estimation for Model 1. Analysis in the case of unknown variance of the measurement noise. The noise is assumed to be additive and the same in all cells. For STS, one estimate of *σ* is obtained from each cell (*blue bars*). **a** For Rich Data, STS can estimate noise through the average of the values (*red line*) equally good as NONMEM (*black line*), even though some individual cells display worse estimates. **b** For the case of Sparse Data, however, the mean from STS is significantly worse than for NONMEM

Next we considered the case of also estimating the measurement noise. All results gave the same type of improvement as for the analysis above. We considered the case where the same noise distribution was used for all cells. In other words, for NLME there was only one additional parameter to estimate: the variance of the noise. For NLME, this means that a single value is obtained for all the cells. Conversely, for STS, which considers the estimation in each individual cell as an independent problem, there were 200 estimated values for this new noise parameter. The results are shown in Fig. 6. As can be seen, NLME performed significantly better in the case of Sparse Data (40 observations per artificial cell), but equally well in the case of Rich Data (240 observations per artificial cell). Again these conclusions are supported by a Student’s t-test (*p*<0.05).

### The results hold for a nonlinear model

We also generated simulated data from the second, nonlinear, model. This generation used a four step input signal (Fig. 4b), simulating the addition of equal amounts of salt four times at equal time intervals. The generated data were divided into four different cases: Original Data, Sparse Data, Noisy Data, and Weak Input Signal Data. The Sparse Data (Fig. 4c and d, black) had 20 observations per output signal, giving a total of 40 observations per artificial cell. The 40 observations can be compared with the 500 observations used in the Original Data (Fig. 4c and d, green). The noise of the Noisy Data (Fig. 4c and d, purple) had a standard deviation of 2.2 compared with 0.5 for the Original Data. The amplitude of the input signal in the data with a weak input signal (Fig. 4c and d, blue) was one tenth of the amplitude of the input signal in the Original Data (Fig. 4b).The parameter estimation with NLME included the true error model and the true, lognormal, form of the parameter distributions.

**Fig. 7:**
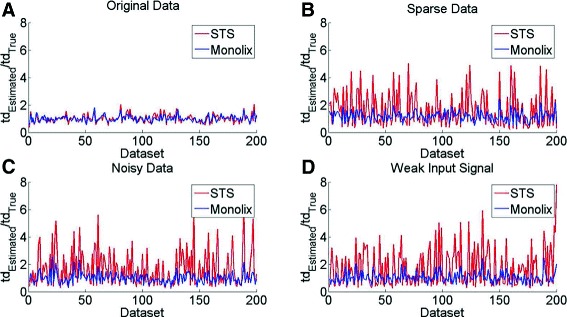
Parameter estimation for Model 2. Analysis in the case of simulated data and parameter estimation for the parameter td in the case of known noise for Model 2 using data that is: **a** under good condition, **b** sparse sampling, **c** noisy, and **d** with a weak input signal respectively. The results are normalized by dividing with the known true value. The x-axes corresponds to the 200 simulated datasets. In comparison, the results from the parameter estimation are similar between STS and Monolix in the case of the Original Data, but there is a clear advantage of using Monolix when the quality of the data decreases

For Model 2, NLME is implemented using Monolix. Figure 7 shows the results of Model 2 for the parameter td. In Fig. 7a, the red and blue curves largely overlap, meaning that in this case, using the Original Data, there is no difference between STS and NLME. In contrast, when the quality of the data is reduced in either of three different ways (sparseness in Fig. 7b, a larger noise level in Fig. 7c, and a weak input signal in Fig. 7d), STS is significantly worse than NLME. These observed differences are confirmed by a paired Student’s t-test (*p*<0.05). Similar results can be seen for the other three parameters, Ve (Figure S2), kp2 (Figure S3), kHOG (Figure S4), in Additional file 3.

### Where does the improvement come from?

The above analysis demonstrates that NLME gives an improvement in the cases of having insufficient information in the data due to either sparsity in sample points, noise or bad input signal leading to bad excitation of the system. This leads to the natural follow-up question of where the improvement comes from. This analysis is done using the JLH approach, and this is done in two ways.

For the first JLH analysis, all parameters are kinetic or initial condition parameters, and are individual to each cell. That means that the joint likelihood function *l*_*tot*_, breaks down into its individual components

(35) $$ l_{\textrm{JLH}}(p^{1},p^{2},\ldots) = l_{1}(p^{1}) + l_{2}(p^{2}) + \ldots   $$

where *l*_*i*_ is the likelihood function considering the data available for the i:th cell only. In other words, in the first JLH analysis, JLH provides no improvement compared to STS, and all the observed improvement comes from the postulation of a joint parameter distribution across the cell population.

For the second JLH analysis, the noise distribution is shared among all cells, meaning that the total likelihood breaks down in the following way

(36) $$ l_{\textrm{JLH}}(p^{1},p^{2},\ldots,\lambda) = l_{1}(p^{1},\lambda) + l_{2}(p^{2},\lambda) + \ldots   $$

where *λ* is the standard deviation of the noise. In other words, using the total likelihood function *l*_JLH_ in eq. (36) for the estimation of all the parameters at the same time, could therefore in principle be an approach that is superior to STS.

**Fig. 8:**
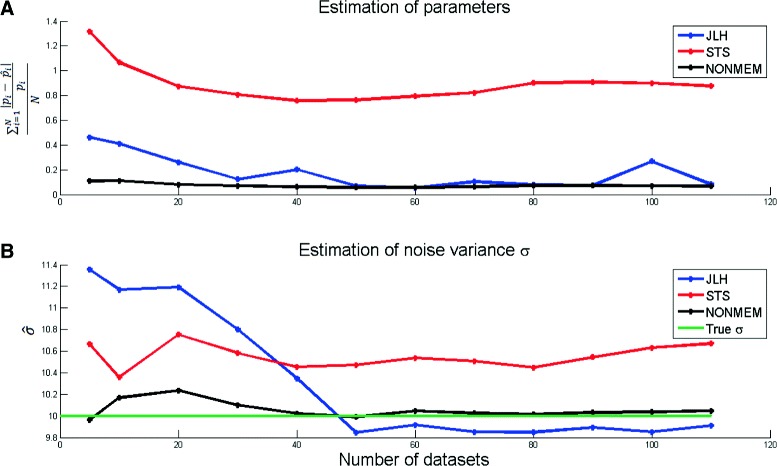
Analysis of STS, NONMEM, and JLH dependency of number of data sets using Model 1. The figure shows STS (*red line*), NONMEM (*black line*) and the JLH approach (*blue line*) precision in the combined parameter and noise estimation problem, with respect to number of datasets. In the JLH approach, a joint likelihood without postulated parameter distributions has been used. **a** the results from the parameter estimation as the normalised sum of the absolute values of the deviation from the true parameter. **b** the results from the noise estimation of true noise level (*green line*). Estimates of both parameters and the noise from both NONMEM and JLH are closer to the true values than estimates from STS. Also, it is clear that NONMEM converge faster towards the true parameters and noise with respect to the number of data sets, when compared to JLH

The result of applying this second approach, in eq. (36), to Model 1 is shown in Fig. 8. As can be seen, a JLH function (blue) does converge faster to the truth for both parameter (Fig. 8a) and noise estimation (Fig. 8b) compared to STS (red). However, JLH is still not as good as NLME (black).

All in all, this means that the improvement of NLME comes purely from the assumption of a shared parameter distribution in cases of only estimating parameters that are unique to each cell; in contrast, the advantage of the shared distribution is combined with the additional advantage of a joint likelihood function, in cases of shared parameters across the cell population (such as the noise in eq. (36)).

### Application to real experimental data

**Fig. 9:**
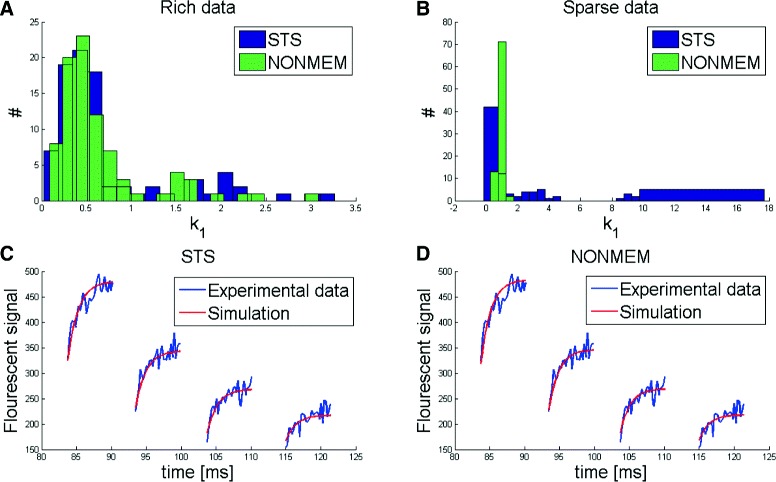
Analysis of STS and NONMEM using real FRAP-data with Model 1. **a** and **b** are histograms of parameter *k*_1_ estimated with NONMEM (*green bars*) and STS (*blue bars*) using all experimental data in each cell (**a**), and only 17 % of the data in each cell (**b**). **a** the parameter distribution from STS and NONMEM coincide in a lognormal distribution. **b**, while NONMEM roughly stays in the same interval as in (**a**), the interval from STS vastly increases. **c** and **d** is a representative example of the model fit to the experimental data from one of the cells using STS and NONMEM, respectively. Results for parameter *k*_2_ shows similar results as for *k*_1_

To demonstrate that NLME is applicable to real data of cell-to-cell variations, we also performed a corresponding analysis for the experimental data analysed using STS and NLME (Methods). Here the true parameters are not known. Using all available experimental data, the estimated parameters from STS and NLME are roughly the same and they both describe the data well as can be seen in Fig. 9a, c, d. However, when removing data from the full time-series, to make the data sparse, new corresponding STS parameter estimates become much more changed than the corresponding NLME estimates, Fig. 9b. This is consistent with the results from the simulated data above.

## Discussion

In this paper, we have answered the questions when, why, and how NLME should be applied to the problem of parameter and noise estimation based on ODE analysis of single-cell data. This analysis brings clear evidence that there are important and common cases when NLME is advantageous compared to the traditional STS approach: in cases of non-informative data, NLME converges faster to the true values.

When considering the case for NLME in single-cell analysis, there are a few strengths that should be further clarified. Firstly, although NLME has only recently been discovered in the analysis of single-cell data, it has a long tradition in other fields, in particular pharmacometrics. Thus, there is already a rich literature of theoretical and methodological results supporting the NLME approach. In other words, the theoretical properties of the method are already well-established, and there are many associated analysis tools that can be used in future analysis of cell-to-cell variation. Secondly, a specific example of such a well-established approach is covariate analysis, which has a high potential also for the study of cell-to-cell variation. Covariate analysis was part of the initial incentive behind the development of NLME: to find correlations between subject-specific characteristics, such as age and weight, with the properties in the model, including subject-specific responses to drug treatments [34]. In the case of cell-to-cell variation, this covariate analysis translates to the identification of correlations between cell-specific characteristics such as cell-line, cell-volume, cell-type, etc, with e.g. the cell-specific kinetic parameters estimated by the model. Thirdly, the rich theory behind NLME also includes estimation of the noise. Such noise estimations are still quite rare in systems biology studies, but are standard in NLME estimations performed in NONMEM. Fourthly, the development of NLME in software packages such as NONMEM and Monolix was driven by challenges commonly seen with patient-specific studies, challenges that also are present in many cell-to-cell variation studies; these packages are thus well adapted for cell-to-cell variation studies. For instance, patient-specific studies often have the limitation that only a few data-points can be collected for each patient; this is a common problem also for cells. Similarly, patient-specific studies are often associated with high noise-levels; high noise-level is also a problem that often becomes pronounced when considering data from individual cells. A final important advantage of NLME is the computational effort. This advantage is primarily seen when comparing NONMEM and JLH of eq. (36). The high computational load of JLH comes from the fact that all parameters have to be estimated in one problem; the computational time in NONMEM and Monolix is reduced via various approximations, which have been developed over the years. NONMEM is also fast because it is implemented in FORTRAN. As an example, the computational time using 100 data sets was roughly 2 hours, and more than 15 hours, for NONMEM and JLH, respectively.

There are naturally also limitations with NLME, and with its current implementations, when considering them in a systems biology single-cell context. For instance, pharmacometrics models are typically small, with around 3–10 states. Today’s single-cell models are usually equally small, but other systems biology models may be substantially bigger, sometimes including hundreds of states. As single-cell omics data becomes increasingly available, this will mean that larger models probably will appear in a single-cell context as well. This will put new challenges to NLME implementations. This challenge is also put forth by the parallel developments of systems pharmacology, which links pharmacometrics models with intracellular models. One other limitation and challenge when adopting NLME to single-cell analysis is the difference in concepts and notions, and also this challenge is also put forth by systems pharmacology. For this limitation, we argue that Monolix is an important alternative to the more widely used NONMEM package, since Monolix is based on Matlab and has a user-friendly interface. Finally, the methodologies considered herein have limitations in terms of their handling of noise, since they do not account for process noise. Therefore, it is important to also follow the implementation of the other sub-communities of NLME, and their implementations into single-cell analysis. The perhaps most important such paper to date is the recent one by Zechner et al. [21].

While the method presented in the Zechner paper is similar to the one we propose in the sense that both methods adapt a mixed-effects approach, there are still fundamental differences. Firstly, the Zechner paper only deals with models based on Continuous-Time Markov Chains. This is a class of models that is fundamentally different from the models based on ODEs that we use. In other words, one cannot simply put a process noise parameter to zero in their formalism and obtain the same equations and methods proposed herein. Secondly, the Zechner paper estimates their parameters using a Bayesian Inference Network, which is an alternative framework, different from the frequentist approaches in NONMEM and Monolix. Finally, we do a thorough analysis of when, why, and how NLME should be applied to single-cell problems. The Zechner paper does not have the kind of convergence and comparison plots that we have (Figs. 5, 6, 7, 8, 9), clearly demonstrating the advantage of NLME compared to STS. Also, the Zechner paper does not unravel the reason why this advantage is there, i.e. they do not differentiate between the different contributions of JLH and the assumption of a joint distribution across the population.

## Conclusions

NLME is a widely used approach in other areas, but it has only recently and in a few papers been applied to single-cell data. No systematic comparison has clearly answered the questions when, why, and how to use it in this new situation. In this paper, we have shown that NLME should be used *when* the available data for each cell are not informative enough to obtain reliable parameter estimates using each cell. If the data are informative enough, NLME provides no advantage in terms of accuracy, but if the data is either too sparse, too noisy, or obtained with a too weak stimulation, NLME has a faster convergence to the true parameter values. This holds for both linear and nonlinear systems, for both simulated and experimental data, and for both parameter and noise estimation. The reason *why* NLME is advantageous is i) that a joint likelihood function is formed, and ii) that one assumes a shared distribution of the parameter values across the cell population. The first factor, i), only contributes if there are shared parameters, such as e.g. the noise level, across the cell population. NLME is implemented in a wide variety of software packages previously not mentioned in the single-cell literature, and we provide a small tutorial for how to use Monolix - a user-friendly and stable alternative - for the analysis of single cell data. This answers the final question of *how* to get started.

## Availability of supporting data

The data sets supporting the results of this article are included within the article and its Additional files.

## Additional files

Additional file 1
**Supporting data.** A zip-file containing the datasets used in the analysis, MATLAB-files, NONMEM-files, and Monolix-files equivalent to the ones used in the analysis, as well as a mini-tutorial of how to use Monolix. (ZIP 1761 kb)

Additional file 2
**Equations for model 2.** A PDF containing the complete equations for the nonlinear model used in the second case study. (PDF 88.8 kb)

Additional file 3
**Supplementary figures and tables.** A PDF with figures showing the results of the estimation of the parameters not shown in the article, as well as tables summarising the results of the Student’s t-tests for the different parameters. (PDF 1085 kb)

## Abbreviations

CTMC: Continous-time markov chain
FRAP: Fluorescent recovery after photo-bleaching
JLH: Joint likelihood function
NLME: Nonlinear mixed-effects modelling
ODE: Ordinary differential equation
PDE: Partial differential equation
ROI: Region of interest
STS: Standard two-stage approach
YFP: Yellow fluorescent protein
