# Supplementary material for: Nonlinear mixed-effects modelling for single cell estimation: when, why, and how to use it
Source: BMC Syst Biol. 2015 Sep 4;9:52. doi: 10.1186/s12918-015-0203-x (PMC4559169; doi:10.1186/s12918-015-0203-x)
Supplement: Additional file 1 — Supporting data. A zip-file containing the datasets used in the analysis, MATLAB-files, NONMEM-files, and Monolix-files equivalent to the ones used in the analysis, as well as a mini-tutorial of how to use Monolix. (ZIP 1761 kb) [file 12918_2015_203_MOESM1_ESM.zip › additional_file_1/monolix_mini_tutorial.pdf]

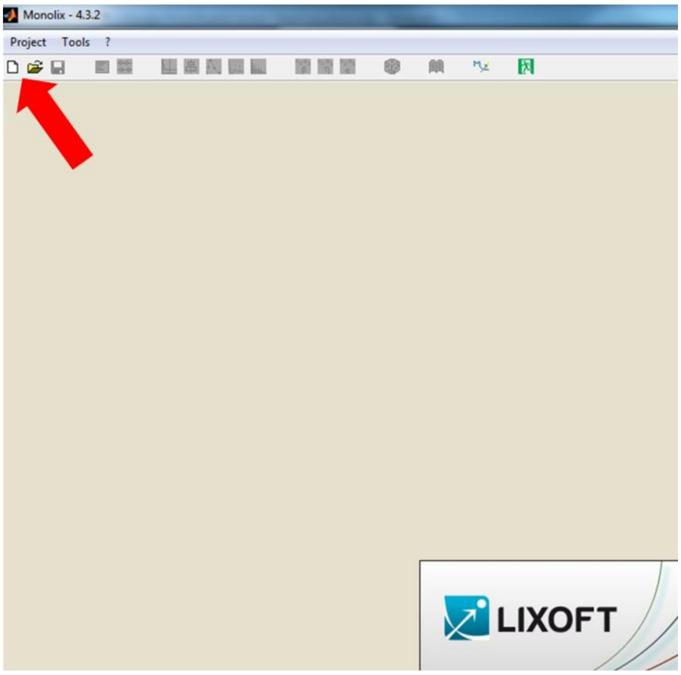

Create a new project.

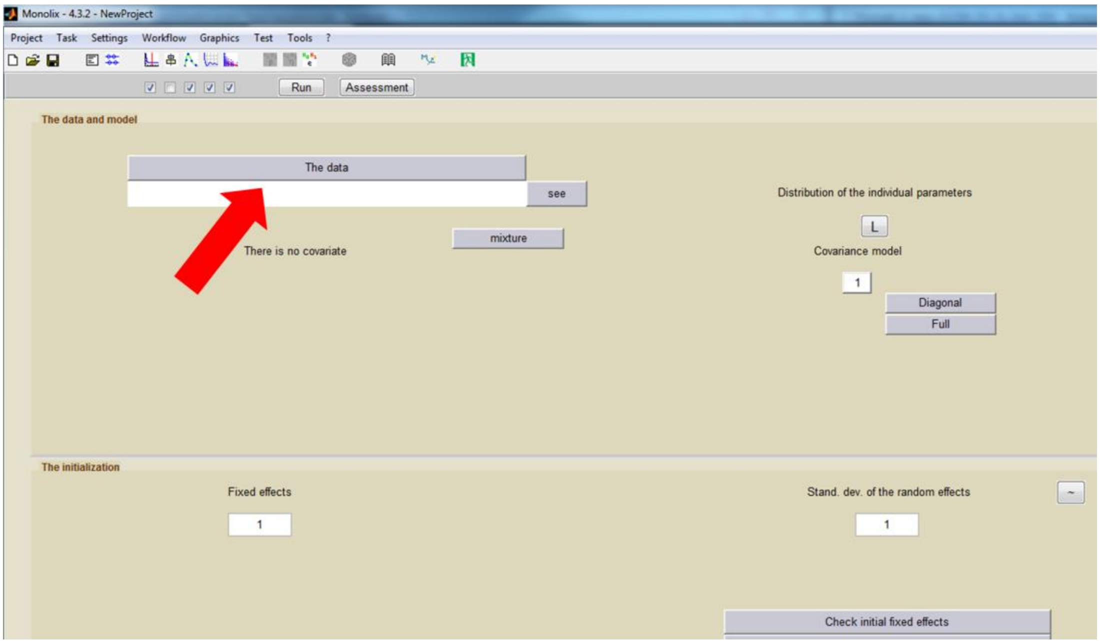

Choose your Monolix data file.

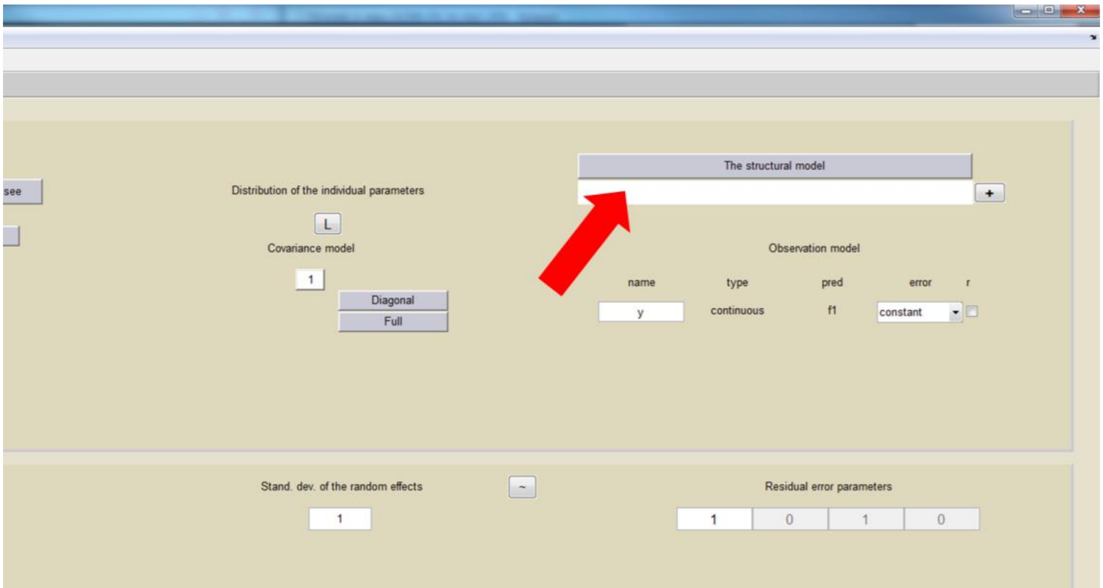

Choose your Monolix model file.

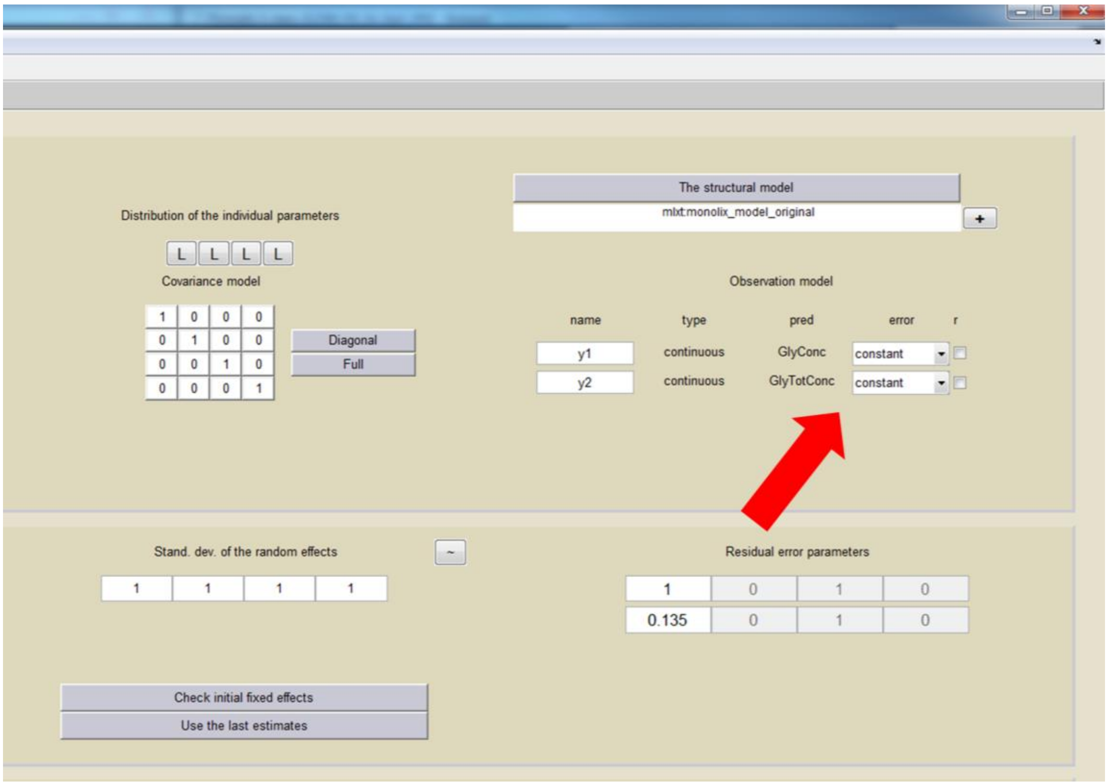

Set the model for the residual errors.

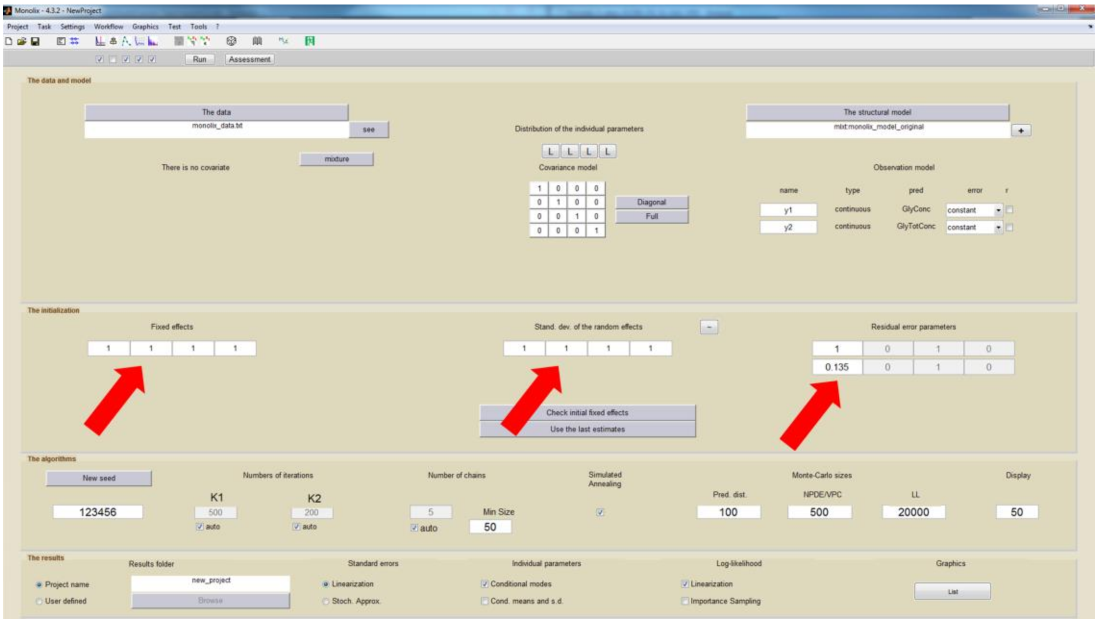

Set initial guesses for population parameters and parameters for the error model.

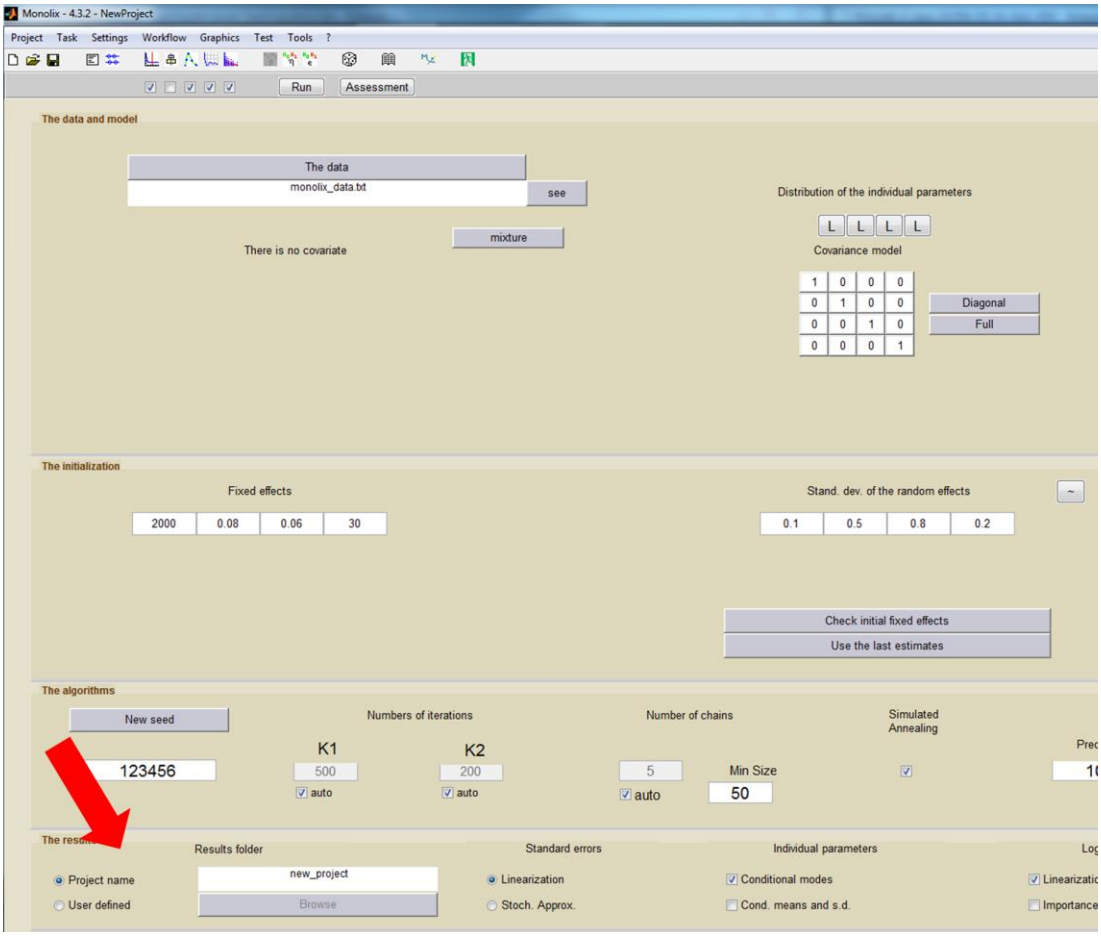

Set the folder where the results should be saved.

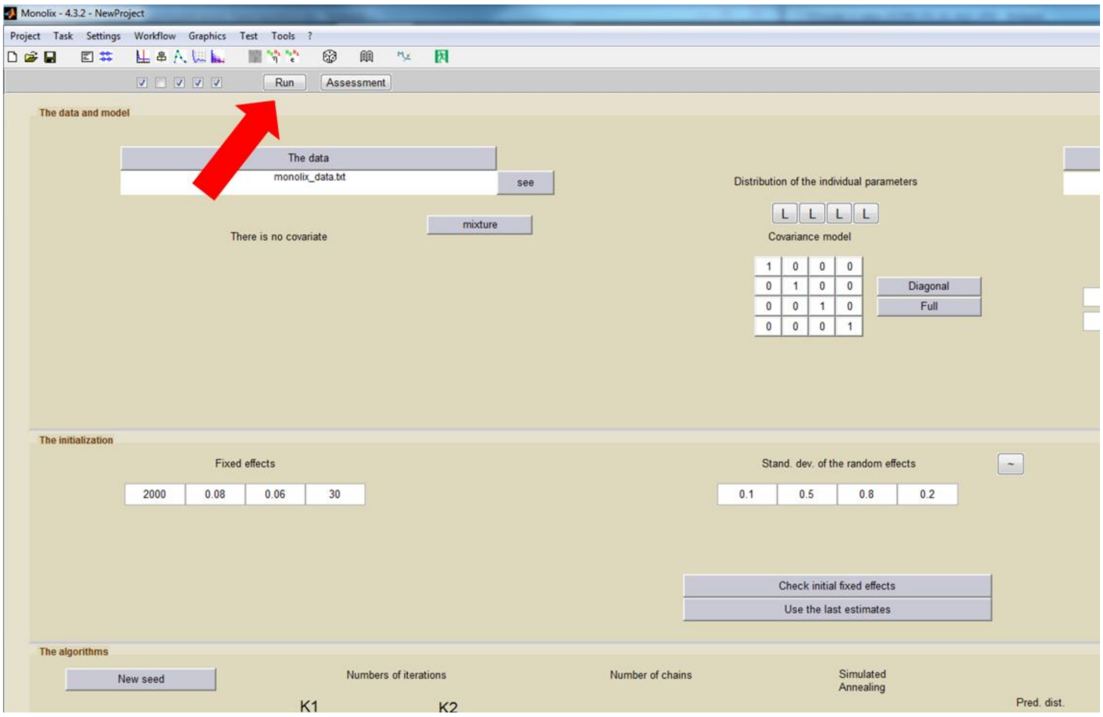

Start the parameter estimation.
