# Supplementary material for: Nonlinear mixed-effects modelling for single cell estimation: when, why, and how to use it
Source: BMC Syst Biol. 2015 Sep 4;9:52. doi: 10.1186/s12918-015-0203-x (PMC4559169; doi:10.1186/s12918-015-0203-x)
Supplement: Additional file 2 — Equations for model 2. A PDF containing the complete equations for the nonlinear model used in the second case study. (PDF 88.8 kb) [file 12918_2015_203_MOESM2_ESM.pdf]

$$\frac{dV}{dt} = k_{p1} \cdot (P_i - P_e - P_t)$$

V is the cell volume, P_i the intracellular osmotic pressure, P_e the extracelluar osmotic pressure, P_t the turgor pressure and k_{p1} the water permeability coefficient.

$$\frac{dGly}{dt} = \upsilon_{HOG} - \mu_{Diff}$$

Gly is the amount of intracellular glycerol, υ_{HOG} the time delayed glycerol production via the HOG pathway and μ_{Diff} the glycerol flow over the Fps1 channel.

$$\frac{dGly_e}{dt} = \mu_{Diff}$$

Gly_e is the amount of extracellular glycerol.

$$\frac{d\upsilon_{HOG}}{dt} = \frac{1}{t_d} \cdot (\mu_{HOG} - \upsilon_{HOG})$$

μ_{HOG} is the undelayed glycerol production and t_d the time delay.

$$P_i = \frac{P_i(0) \cdot (V(0) - V_b) - Gly(0) + Gly}{V - V_b}$$

$P_i(0)$ is the initial intracellular osmotic pressure, V(0) the initial cell volume, V_b the non-osmotic volume of the cell and Gly(0) the initial amount of intracellular glycerol.

$$P_t = \frac{(P_i(0) - P_e(0)) \cdot (V - V_t^{P=0})}{V(0) - V_t^{P=0}}$$

$When\ V > V_t^{P=0}\ otherwise\ P_t = 0$

P_e is the initial extracellular osmotic pressure, ${V_t}^{P=0}$ the cell volume when P_t=0.

$$P_e = P_e(0) + P_{Stress}$$

P_{Stress} is the added osmotic pressure, i.e. the input signal.

$e = P_t(0) - P_t$

e is the input signals to the proportional controllers and $P_t(0)$ the initial turgor pressure.

$$\mu_{Fps1} = \frac{k_{p2} \cdot (P_t(0) - e)}{P_t(0)}$$

$When\ e > 0\ otherwise\ \mu_{Fps1} = 0$
k_{p2} is the glycerol permeability coefficient in a completely open Fps1 channel

$$\mu_{Diff} = \mu_{Fps1} \cdot \left(\frac{Gly}{V - V_b} - \frac{Gly_e}{V_e}\right)$$

V_e is the extracellular volume.

$$\mu_{HOG} = k_{HOG} \cdot e$$

$When\ e > 0\ otherwise\ \mu_{HOG} = 0$
k_{HOG} is the proportional control constant for the production of glycerol via the HOG pathway.

$$GlyConc = \frac{Gly}{V - V_b}$$

Measurement equations, concentration of intracellular glycerol.

$$GlyTotConc = \frac{Gly + Gly_e}{V - V_b + V_e} \cdot 1000$$

Measurement equations, concentration of total glycerol.

$V(0) = 1.00$
$Gly(0) = 2.00 \cdot 10^{-4}$
$Gly_e(0) = 1.52$
$\upsilon_{HOG}(0) = 0$
$V_t^{P=0}) = 0.99$
$P_e(0) = 0.24$
$P_i(0) = 0.64$
$V_b = 0.37$
$k_{p1} = 1$
