# Supplementary material for: Nonlinear mixed-effects modelling for single cell estimation: when, why, and how to use it
Source: BMC Syst Biol. 2015 Sep 4;9:52. doi: 10.1186/s12918-015-0203-x (PMC4559169; doi:10.1186/s12918-015-0203-x)
Supplement: Additional file 3 — Supplementary figures and tables. A PDF with figures showing the results of the estimation of the parameters not shown in the article, as well as tables summarising the results of the Student’s t-tests for the different parameters. (PDF 1085 kb) [file 12918_2015_203_MOESM3_ESM.pdf]

Fig S1, Karlsson et al

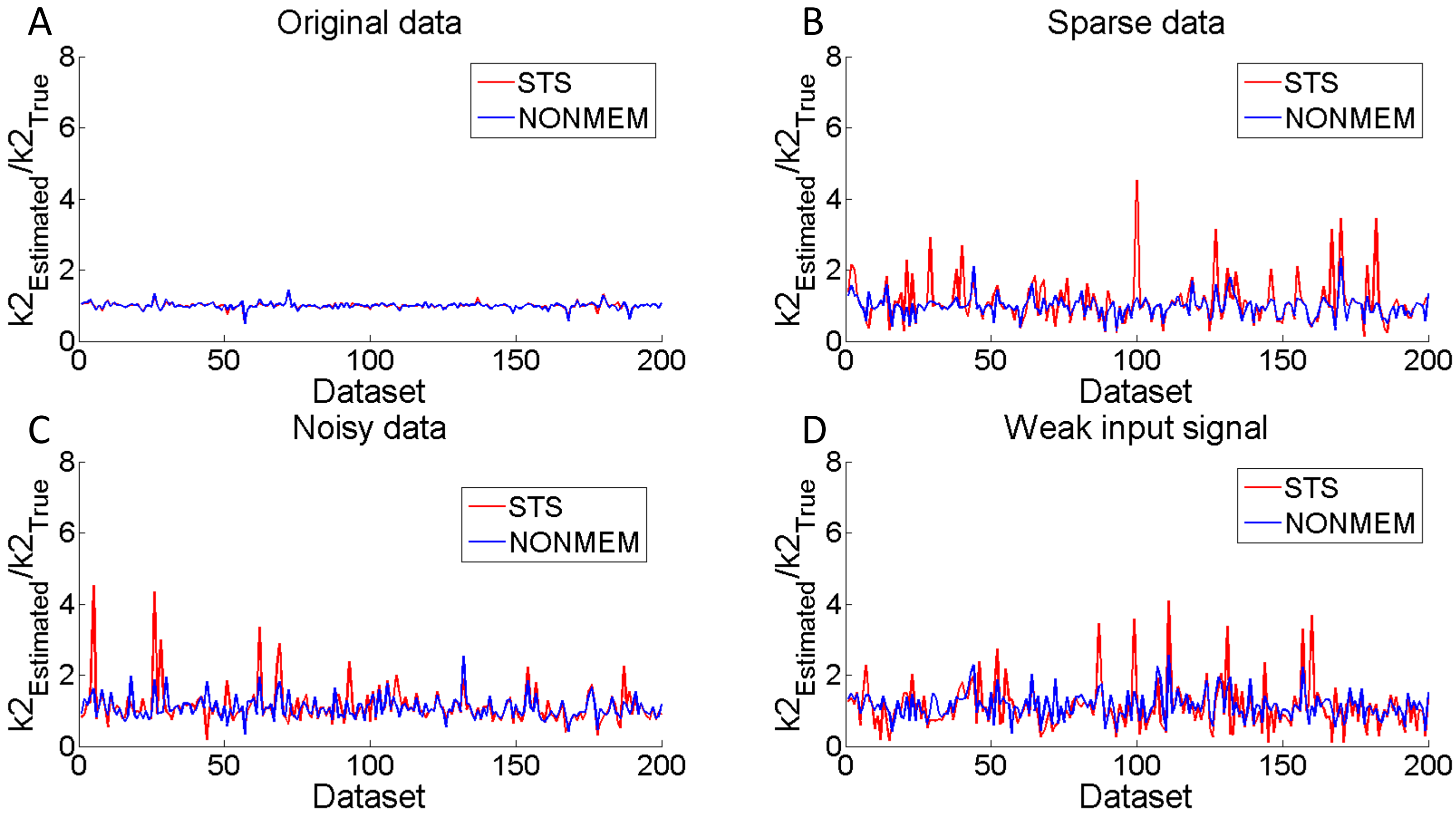

Fig S1: Analysis in the case of simulated data and parameter estimation for the parameter k_2 in the case of known noise for Model 1 using data that is: (A) under good condition, (B) sparse sampling, (C) noisy, and (D) with a weak input signal respectively. The results are normalised by dividing with the known true value. The x-axes corresponds to the 200 simulated datasets. In comparison, the results from the parameter estimation are similar between STS and NONMEM in the case of the Original Data, but there is a clear advantage of using NONMEM when the quality of the data decreases.

Fig S2, Karlsson et al

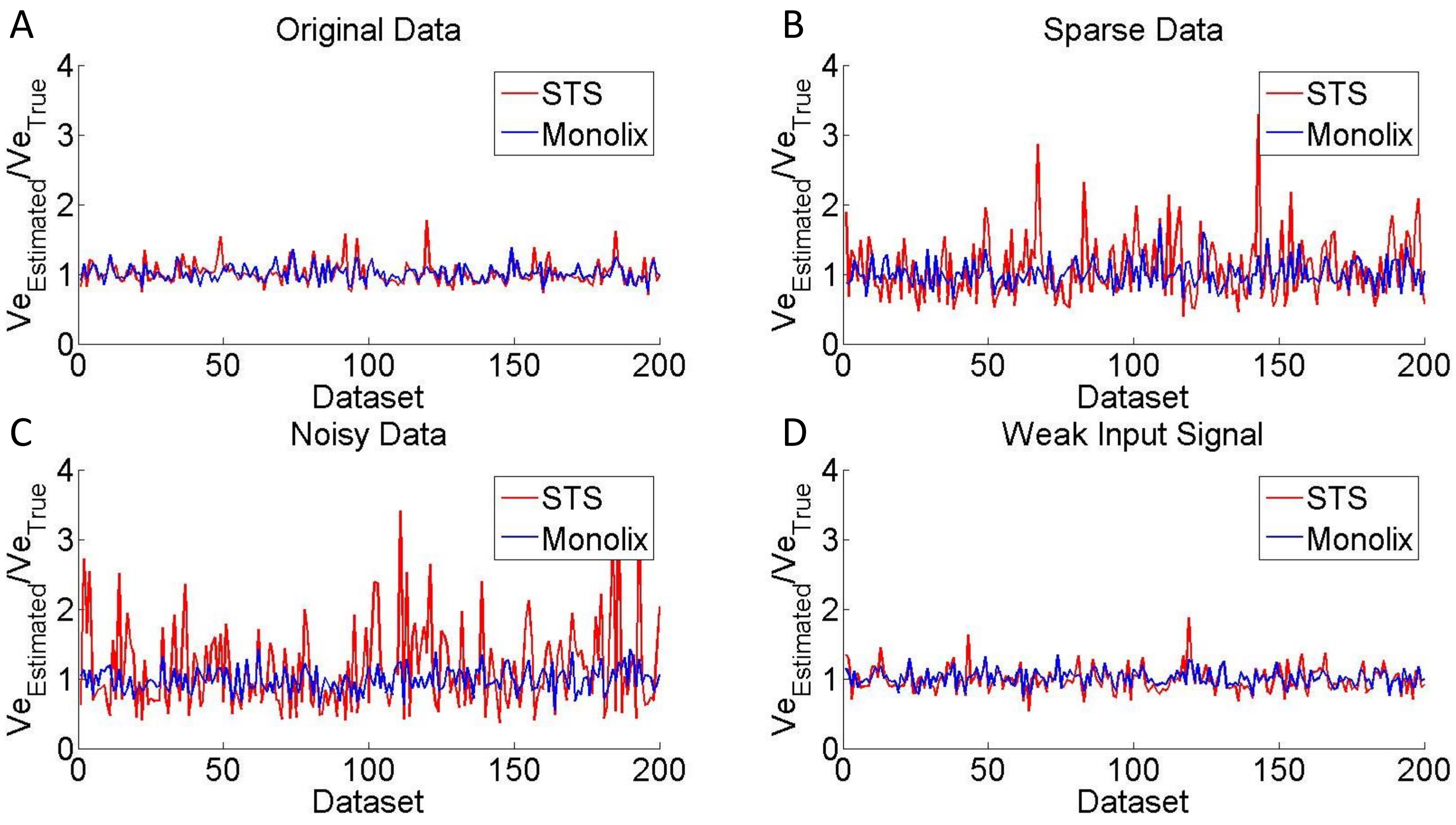

Fig S2: Analysis in the case of simulated data and parameter estimation for the parameter Ve in the case of known noise for Model 2 using data that is: (A) under good condition, (B) sparse sampling, (C) noisy, and (D) with a weak input signal respectively. The results are normalised by dividing with the known true value. The x-axes corresponds to the 200 simulated datasets. In comparison, the results from the parameter estimation are similar between STS and Monolix in the case of the Original Data, but there is a clear advantage of using Monolix when the quality of the data decreases.

Fig S3, Karlsson et al

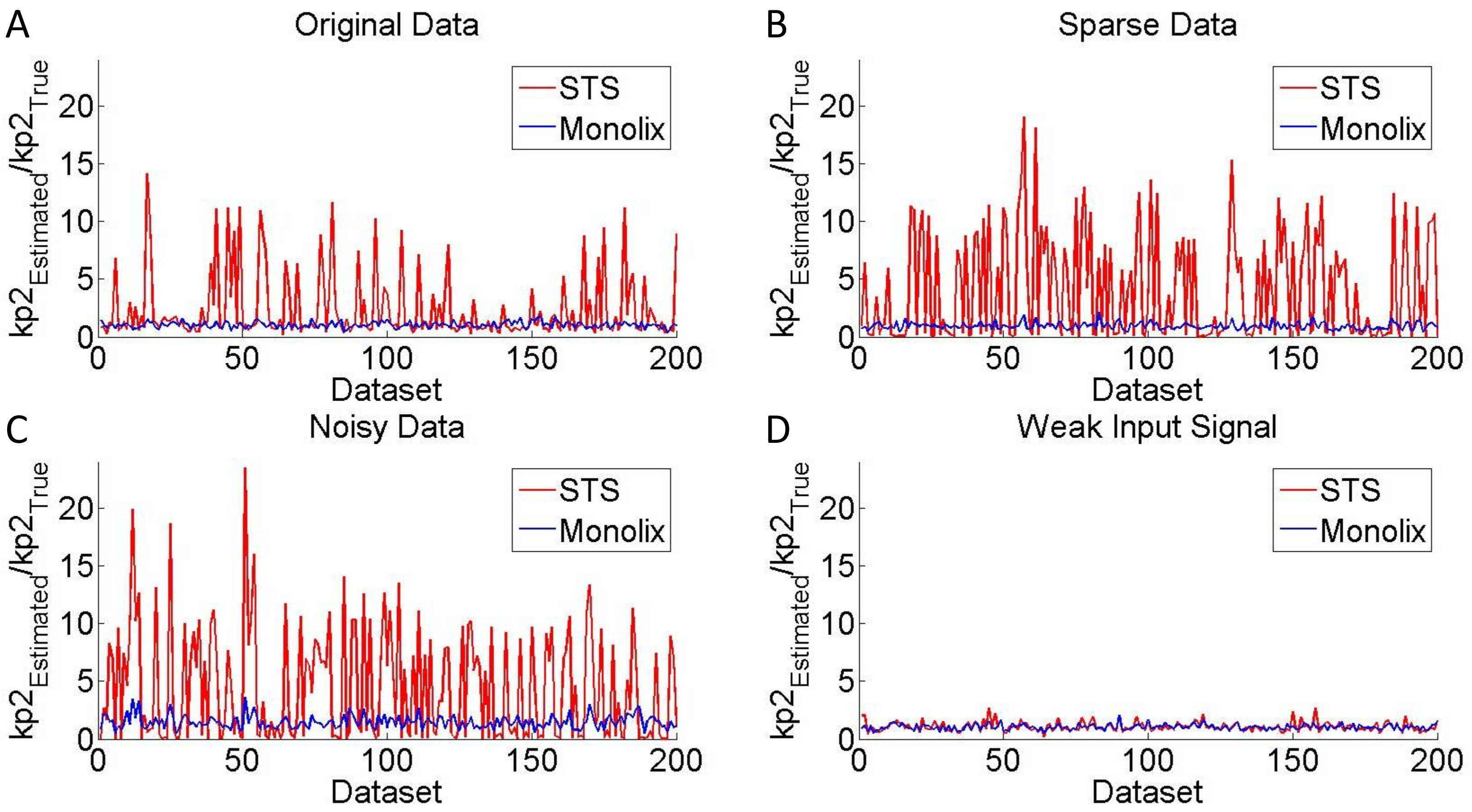

Fig S3: Analysis in the case of simulated data and parameter estimation for the parameter kp2 in the case of known noise for Model 2 using data that is: (A) under good condition, (B) sparse sampling, (C) noisy, and (D) with a weak input signal respectively. The results are normalised by dividing with the known true value. The x-axes corresponds to the 200 simulated datasets. In comparison, the results from the parameter estimation are similar between STS and Monolix in the case of the Original Data, but there is a clear advantage of using Monolix when the quality of the data decreases.

Fig S4, Karlsson et al

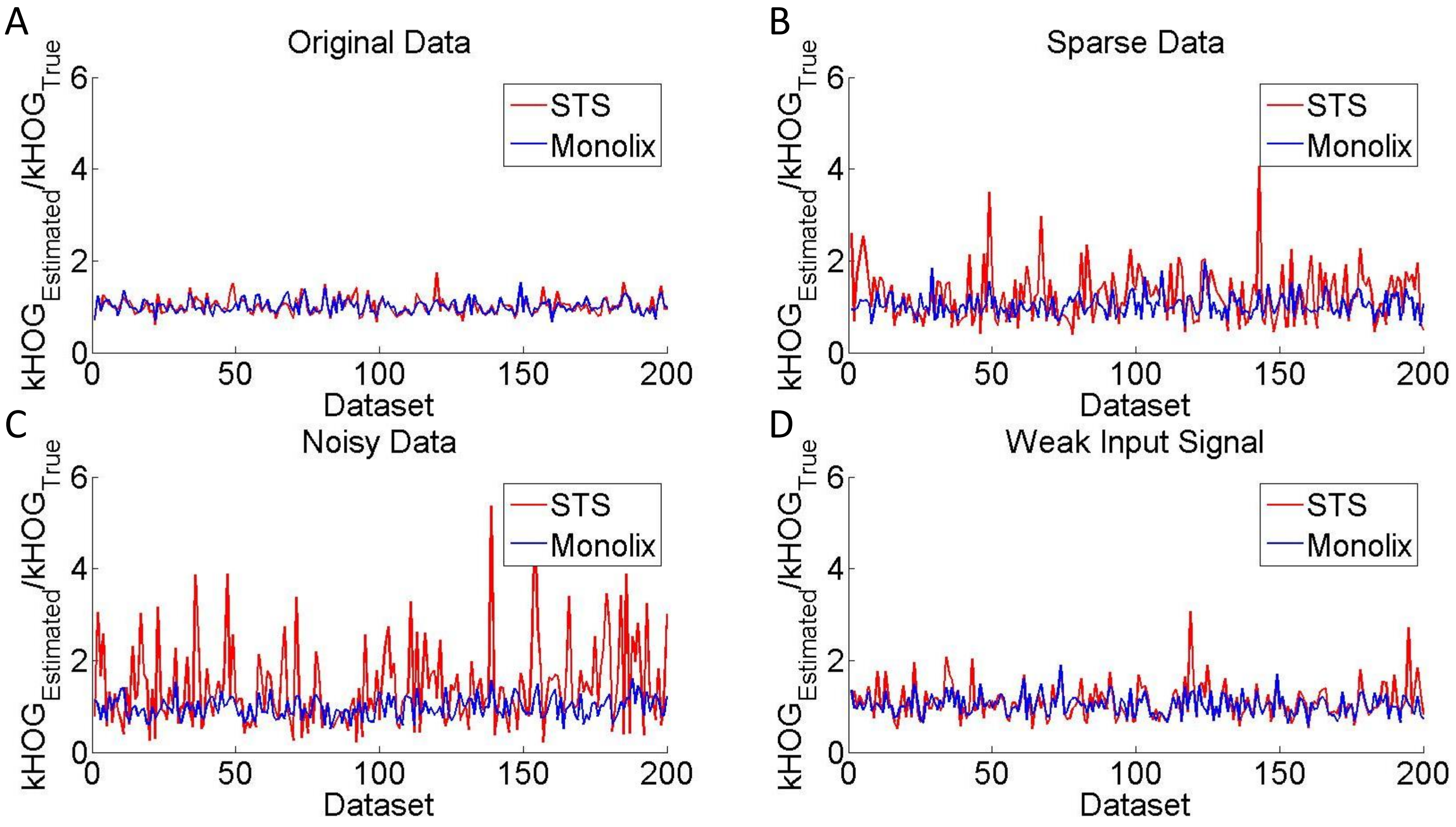

Fig S4: Analysis in the case of simulated data and parameter estimation for the parameter kHOG in the case of known noise for Model 2 using data that is: (A) under good condition, (B) sparse sampling, (C) noisy, and (D) with a weak input signal respectively. The results are normalised by dividing with the known true value. The x-axes corresponds to the 200 simulated datasets. In comparison, the results from the parameter estimation are similar between STS and Monolix in the case of the Original Data, but there is a clear advantage of using Monolix when the quality of the data decreases.

	k1	k2
original	o	x
sparse	x	x
noisy	x	x
weak	x	x

Table 1: Table S1: A summary of the results from the Student t-test analysis of the estimation parameters in Model 1. x = significant difference between the results from STS and NONMEM, o = no significant difference ($p < 0.05$).

	Ve	kp2	kHOG	td
original	o	x	o	o
sparse	x	x	x	x
noisy	x	x	x	x
weak	x	o	x	x

Table 2: Table S2: A summary of the results from the Student t-test analysis of the estimation parameters in Model 2. x = significant difference between the results from STS and Monolix, o = no significant difference ($p < 0.05$).

Comments on Supplementary Figures and Tables

The four figures (S1-S4) shows the rest of the omitted parameter estimation results. As described in the paper, the parameter estimation was performed under the following four conditions where the data was information rich, sparse, noisy and generated with a weak input signal respectively. For a summary of all Student t-tests for the parameter estimation see Table S1 and S2.

In general, NONMEM/Monolix outperforms STS under all conditions where the data is degenerated for all model parameters. The exception is for the parameter kp2 where Monolix only outperforms STS with information rich data, sparse data and noisy data but not with the data set generated using a weak input signal. This might seem counter intuitive. However, while there is no doubt that by having sparse or noisy data means less information, the same argument cannot in general be said about the input signal. For nonlinear systems, such as Model 2, whether or not the systems gets sufficiently excited by the input signal depends both on the model structure and the characteristics of the input signal such as time profile and frequency.

In other words, simple varying the amplitude of a input step signal to a nonlinear system does not by default result in more or less excitation of the system. With this reasoning in mind, a possible explanation to the behavior of kp2 is could be that a step function as a input signal with a lower amplitude excites the system more than compared to a step function as an input signal with a higher amplitude.
